# Necrosis of the Lower Jaws

**Published:** 1871-08

**Authors:** D. W. Clancey


### NECROSIS OF THE LOWER JAWS.

BY D. W. CLANCEY, M. D., D. D. S.

A short time since, Mr. H , a young man of about twenty years of age, was directed to consult me in regard to his lower maxilla. He had lost the cuspid, central and lateral incisors, and the remaining central was quite loose, from what he supposed to be the effects of tartar. I made an appointment with him for the following morning, telling him what I supposed to be the trouble, and that it would be necessary for him to submit to an operation, to which he cheerfully consented, and was on hand to meet his engagement.

Upon close examination, I found it to be a well marked case of necrosis. The central incisor remaining could be moved quite easily, but gave a sense of crepitation to the touch. The extent of the lesion was from twelve to fourteen lines in length, commencing at the mental foramen and passing toward and by the symphysis, the sequestrum just visible in one or two places through the fleshy parts, and emitting a rather sanious discharge, making him exceedingly disagreeable, both to himself and his friends.

After assuring myself of the extent and condition of the case, I provided myself with a curved bistoury, pliers, and a pair of process forceps. Separating the soft parts on a line with the jaw, and grasping the dead bone with the forceps, it came away without any trouble, bringing the loose tooth with it, and with very little shock to the patient. By using alum water, the loss of blood was very slight.

The man's appearance aroused my suspicion that the necrosed bone was the result of a dyscrasia of the blood, so I made an examination of the entire dental arches, taking each tooth separately, and found the right central and lateral superior incisors, giving a very slight crepitation, and having a "numb feel," as the patient called it. In appearance the teeth seemed quite as healthy as their fellows on the opposite side; the gums over these teeth were not swollen to any appreciable amount, and only a little darker than the contiguous parts, and I found it difficult to convince him there was anything wrong, until I made pressure on the surface, and a slight discharge was emitted from under the free borders of the gum.

The wound left after removing the offensive mass; was dressed with a pellet of raw cotton, saturated in a weak solution of carbolic acid a few times, and it healed in a short time.

Patient admitted he had taken calomel in considerable quantities, but denied positively that he had been salivated when I explained the symptoms. However, I am inclined to regard it a case of mercurial necrosis, and though the affected portion of the superior maxilla was injected with elixor vitriol and iodine, and the gums with the internal use of nitric acid, with a hope of restoration, yet I am inclined to think that nature will throw off the affected portion of the bone by way of self-protection under any treatment, where the disease has made even the apparently slight advances that this case has.

While there is nothing very marked in this, that is not peculiar to necrosis in this situation, I have been induced to report it because I feel that contributors to our dental periodicals are painfully silent as to their experience in the management of diseases and injuries of the facial bones and contiguous parts. It may be claimed by some that when we go beyond the teeth, we overstep the bounds of our province as dental surgeons, and should turn our patient over to the physician and surgeon, who is expected to cope with every malady that flesh is heir to, no matter in what part of the body it occurs, thus giving our patient, his friends, (and the doctor, too, for that matter,) the impression that they are not to think of a dentist again, only in connection with a toothache. This view of the matter may be extreme, and I know is not the experience of some few, but to be candid, I would ask every member of the profession whose heart is for its true elevation, if this is not the tendency on the part of communities? Why is this? Why is it that as a general thing, the dental surgeon is so rarely consulted in those cases where any portion of the mouth, except the teeth, are involved, or in short, anything, that in the opinion of the patient, requires the services of a *surgeon?* Is it because it is tacitly understood that his knowledge of the human system is so *limited* and *superficial* that he has no just claim to the title of *surgeon*, but has appropriated and by common consent, used it to add weight and dignity to his own calling? If this is so, the blame rests with himself and his class, and no one else. Communities, like individuals, are what they are educated to be, and until the aspiration becomes more general on the part of the profession to do something more than "good jobs, good fits—work that will *stand*," etc., (all of which are quite essential so far as they go,) the dentist will not be called on so far as surgery goes, much beyond the extraction of a tooth, notwithstanding our dental colleges, hospital clinics, and dissecting.

I see no reason why the dentist should not stand on the same level with the physician in regard to surgery, if any difference, circumstances favor *him* rather than the latter. His superior skill in the use of instruments and his mechanical ingenuity alone would recommend his hand to be intrusted with the scalpel.

Some may contend that the study of the pathology of the dental tissues alone, to its utmost prosecution, is beyond the grasp of an ordinary mind. I would inquire of such, in what respect on general principles, does the phenomena of disease in this part of the economy differ from the whole, that the treatment should be a "law unto itself;" that the principles are not given in any standard text book on surgery, we may take up, to be carried out by an *enlightened* judgment.

If facts will not bear me out in this, then it may be inferred that the physician's and surgeon's diagnosis and prognosis of his cases, as they come up, can not but be unreliable, and thus life and health are in constant jeopardy all over the civilized world. I think that there are few, no matter how warmly they advocate the *exclusive* study of dentistry, that after mature consideration, would have the boldness to make such an assertion, and yet we must accept it in this light, if we adhere to the first proposition, or make an admission that would not be at all complimentary to us as a class. That is, that naturally in the main; we choose our specialty, because of a consciousness of our mental inferiority, and timorously pause on the threshold of medical and surgical science, doubting our capacity to enter in.

That as a class, the young men of the present, entering on the study of dentistry, are naturally inferior to those engaged in the study of medicine, I most emphatically deny, for if we take the trouble to visit the different medical schools throughout the country, and compare them with our dental schools, we will find the mental power of the student in each will average about the same. And as they both proceed from the college halls to their respective fields of labor, if the physician has placed in his hand a higher trust, it is because his studies anticipate that trust, and not from the inferiority of the man.

Dr. Homer Judd, of St. Louis, at the last annual session of the Missouri State Dental Association, made an assertion, that one half of those now engaged in the practice of dentistry througout the country, are "totally ignorant" of its commonest principles, and over half of the remainder are incapable of performing the simplest operations with benefit to their patients." This is not very flattering to men claiming to have a liberal profession, and yet, coming from the source it does we can hardly doubt its correctness.

I commenced these remarks on the hypothesis that every dentist should be a surgeon *par excellence*, and hold his place in the estimation of the public as such. A man that would be as capable of ligating an artery as he would be of adjusting the "dam" preparatory to filling, or of operating for stone in the bladder, as he would be of extracting a tooth. These attainments are available to all who have the ambition, and surely, he who would take upon himself the responsibilities of the grave profession of ministering to health and life of his suffering fellow creatures, ought to be susceptible of the very highest culture. The public will, in most cases, discriminate between such a one and the pretender, who learns his calling as a mere trade. That the latter belongs to much the largest class is evident, and the amount of prejudice to overcome in reaching a higher plane is proportionately great, is no reason why the man of science acquired by patient and continued investigation, should allow himself to be identified by his community with any scalawag, who happens to be prowling about, having made a shift at the business to get his bread and butter.

Perhaps some one of the readers of the REGISTER has a blessing or two of this kind in your neighborhood, in the shape of a "resident dentist," who "puts in nice teeth," and "knows all about it," and you are mortified occasionally by one of the Doctor's (?) "customers," (who has become convinced that he has been imposed upon,) who opens his mouth at you, and invites you to "take a look," the first time he meets you in a public place. When you remind him that he may consult you at your office, at his earliest convenience; that you have no desire and are not prepared to make an examination in such a place, he will say, "perhaps, that would be better, where is your shop?" Though you are chagrined over this little scene, you can not blame this person, he has just as high an estimation of dentistry as a profession, as he has of tailoring or shoemaking; it is a matter of education with him, and his conduct towards you is but the result of his impression of his former dentist, and not because he is inclined to be disrespectful to you.

It is true that dentistry has been very progressive within the last few years, and equally true that quackery has kept pace. Aye more! They have out-quacked quackery—they do it by steam (!) since the rubber dispensation.

How is all this to be changed? "That is the question." a practical solution of which would be hailed by many throughout the country with unbounded satisfaction. Now if we wish to lose our identity with those persons now engaged in mounting artificial teeth and boiling rubber, we can only find an exodus through a more liberal medical education, which is at our door, and leave them to higgle over and settle their prices between themselves. More anon.

